# The Molecular Links of Re-Emerging Therapy: A Review of Evidence of Brahmi (*Bacopa monniera*)

**DOI:** 10.3389/fphar.2016.00044

**Published:** 2016-03-04

**Authors:** Deepali Mathur, Kritika Goyal, Veena Koul, Akshay Anand

**Affiliations:** ^1^Department of Functional Biology, Faculty of Biological Sciences, University of ValenciaValencia, Spain; ^2^Neuroscience Research Lab, Department of Neurology, Post Graduate Institute of Medical Education and ResearchChandigarh, India; ^3^Center for Biomedical Engineering, Indian Institute of TechnologyNew Delhi, India

**Keywords:** Brahmi, pharmacological effects, anti-parkinson, anti-convulsant, anti-depressant, clinical trials

## Abstract

The convolution associated with memory is being resolved with advancement in neuroscience. According to the concurrent assumptions, synaptic plasticity forms one of the basis of memory formation, stabilization and strengthening. In Alzheimer's disease (AD), which is generally characterized by memory dysfunction, connections amongst the cells in the brain are attenuated or lost leading to degeneration of neural networks. Numerous attempts have been made to find new therapies for memory dysfunction with increasing attention and investments being laid on herbal drugs. Many herbal plants and extracts have already documented beneficial results when tested for antiamnesic effects. Brahmi (*Bacopa monniera*) is one such common herbal drug, which is employed for a long time in the Indian and Chinese medical system in order to treat several disorders. Previous research has shown that Brahmi exerts many pharmacological effects including memory boosting capacity in the treatment of Alzheimer's disease and Schizophrenia, exhibiting antiparkinsonian, antistroke, and anticonvulsant potentials. The present review discusses the chemical constituents of Brahmi along with *in vitro* and *in vivo* studies based on the pharmacological effects exerted by it. The efficacy of Brahmi in treating various disorders has evoked sufficient research in recent years and now it is a time to launch multiple clinical trials.

## Introduction

The significance of Brahmi (*Bacopa monniera* Linn.) in improving memory and learning skills was first published in 1982 (Singh and Dhawan, 1982). Since then various studies have been conducted in animals to determine various properties exhibited by the medicinal herb. The potential of Brahmi in shielding neuronal structure and/or function has also been evaluated in a number of growing studies. Brahmi is a well-known Ayurvedic medicinal herb, which is re-emerging as a recourse to treatment of memory related disorders. Its medicinal potency is reported both in Indian as well as Chinese traditional literature. Although many chemical compounds have been isolated from Brahmi, the active fractions of this medicinal plant contain bacoside-A and bacoside-B. A number of other phytochemicals such as alkaloids, glycosides, flavonoids, saponins etc. are the constituents of Brahmi (Dutta and Basu, 1963; Chatterji et al., 1965; Basu et al., 1967).

**Figure 1:**
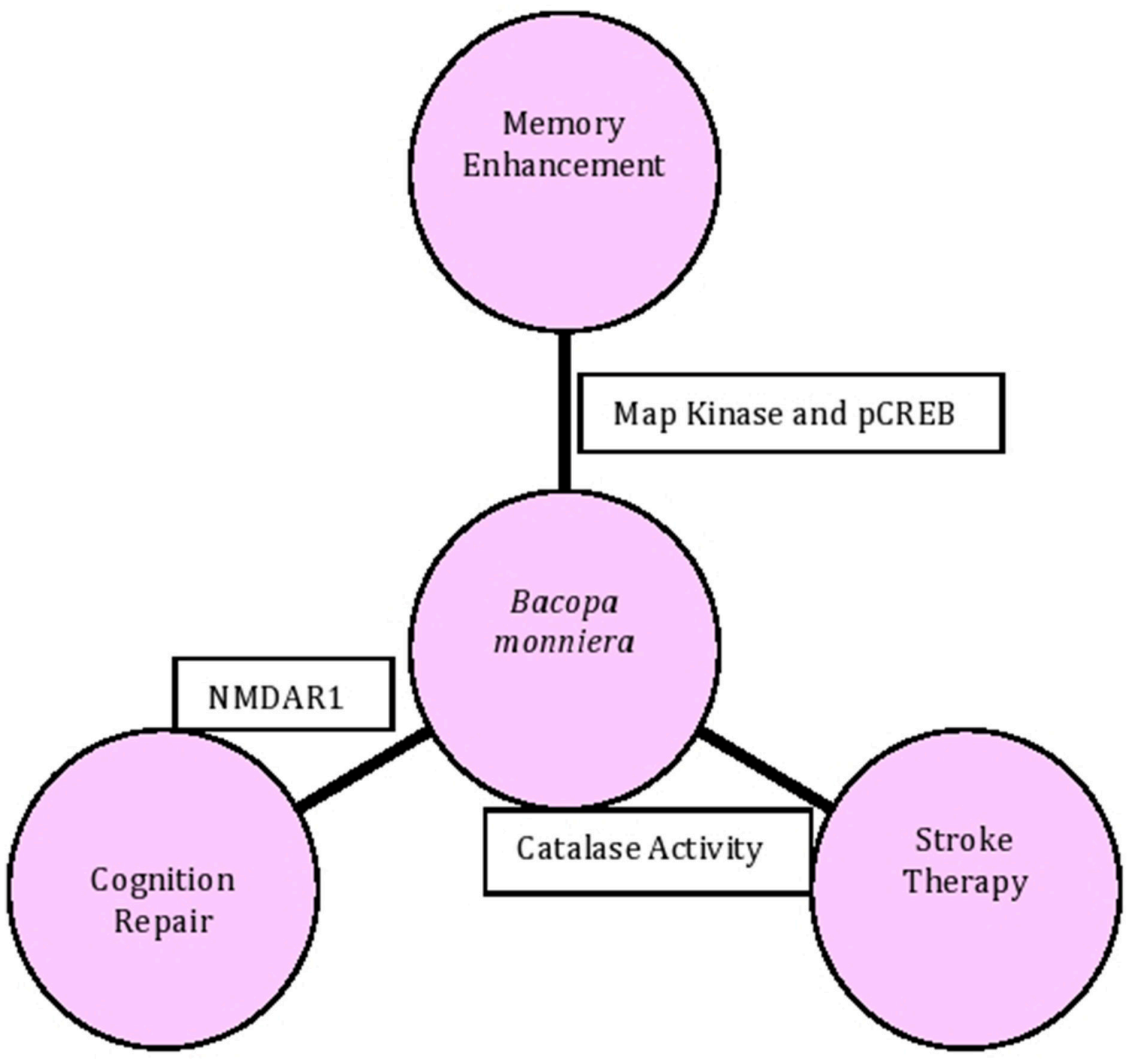
**Major pharmacological effects of Brahmi with respective target molecules**.

Investigations conducted so far have revealed that Brahmi exerts many pharmacological effects (Figure 1) including memory boosting effect in the treatment of Alzheimer Disease and Schizophrenia, besides displaying antiparkinson, antistroke, and anticonvulsant potentials. The present review discusses the chemical constituents of Brahmi together with *in vitro* and *in vivo* studies based on its molecular and pharmacological effects (Figure 1).

## Chemical constituents of brahmi

**Figure 2:**
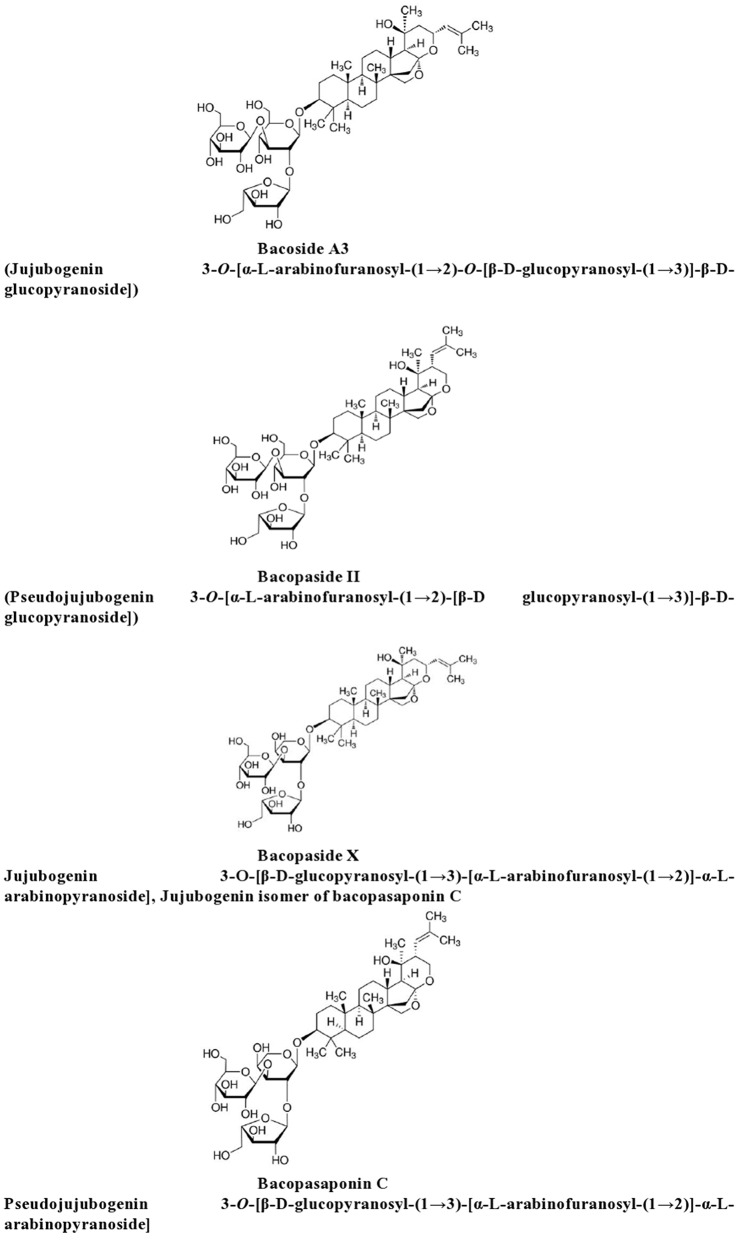
**Chemical constituents of Brahmi**.

*Bacopa monniera* is characterized by its typical chemical composition which predominantly includes compounds like dammarane-type triterpenoid saponins called as bacosides, with jujubogenin or pseudo-jujubogenin moieties as their aglycone units. Based on the structural similarity, 12 analogs from the family of Bacosides have been elucidated. In the recent past, bacopasides I–XII, a different class of saponins have been identified as an important constituent of the herbal extract (Rauf et al., 2013). Apart from hersaponin, apigenin, D-mannitol, monnierasides I-III, plantainoside B and cucurbitacin; the alkaloids brahmine, herpestine and nicotine have also been classified in the chemical constituents of *Bacopa monniera*. Bacoside A is the most studied and potent constituent of Bacopa, which is composed of bacoside A3, bacopasaponin C, bacopaside II and bacopaside X (Srivastava et al., 2012; Deepak and Amit, 2013; Singh et al., 2014; Figure 2).

Various research groups have separated the constituents of Brahmi through HPLC. Several mobile phase systems have been used for the purpose of separating various chemical constituents of Brahmi like methanol and water (Ganzera et al., 2004); mixture of acetonitrile and 0.05 M sodium sulfate (pH 2.3; 68.5:31.5; Sivaramakrishna et al., 2005; Murthy et al., 2006); 0.25% orthophosphoric acid in water and acetonitrile (Phrompittayarat et al., 2007; Sumathi and Devaraj, 2009); but till now no consensus mobile phase system has come up for the analysis of Brahmi constituents.

## Active components

The therapeutic effects of Bacopa monniera are believed to be exerted through triterpenoid saponins present in the plant extract. Bacosides are the triterpenoid saponins of prime importance. They have been shown to enhance nerve impulse transmission. The bacosides promote the repair of damaged neurons by upregulating neuronal synthesis and kinase activity. The bacosides also aid in the restoration of synaptic activity, which ultimately leads to nerve impulse transmission (Singh and Dhawan, 1997). The nerve impulse transmission, plays a vital role in promoting healthy cognitive functions like attention span, focus, concentration, learning and memory. There is evidence which suggests that Bacopa, by the virtue of containing active constituents like bacosides, influences the synthesis and availability of the neurotransmitter, Serotonin; therefore, Bacopa helps to maintain neurotransmitter balance (Charles et al., 2011; Rauf et al., 2012a).

## Pharmacological effects

### Memory booster in Alzheimer's disease and schizophrenia

Brahmi *(Bacopa monniera)* has been used in the form of memory enhancer for many years. The accreditation of the traditional assertion of Brahmi was initiated by investigating the effect of an alcoholic extract of this herb on acquisition, consolidation and retention in different conditioning schedules in rats. These included shock driven brightness-discrimination response, continuous avoidance and active conditioned response. It was found that motor skills, acquisition and consolidation were improved and newly acquired behavior was retained for a long period of time in all the three learning responses by the introduction of the CDRI-08 (KeenMind; 40 mg/kg, po. × 3d) in mice (Singh and Dhawan, 1982, 1997). To discern the efficacy of Brahmi in causing the reversal of amnesia, several behavioral studies have been conducted by inducing amnesic agents in animals. Some of the potential amnesic agents including benzodiazepines, scopolamine, quinoline derivatives and phenytoin cause amnesia by interrupting long-term potentiation (LTP). The process of LTP is probably interfered by the involvement of gamma-aminobutyric acid-benzodiazepine pathway. Saraf et al. demonstrated that amnesia induced by diazepam (1.75 mg/kg) was significantly reversed by Brahmi (120 mg/kg) which was provided orally in mice (Prabhakar et al., 2008). Subsequently, the same group later examined the influence exerted by Brahmi on the downstream signaling molecules related to LTP in amnesic mice, which were developed by diazepam (Saraf et al., 2008). The molecular tests revealed that diazepam upregulated the gene expression of inducible nitric oxide synthase (iNOS), mitogen activated protein kinase (MAP kinase) and phosphorylated CREB (pCREB) whereas reduced the expression levels of cAMP response element binding protein (CREB), cyclic adenosine monophosphate (cAMP), total nitrite and nitrate. The levels of calmodulin remained unaltered with diazepam induction. On the contrary, administration of Brahmi inhibited the increased expression of iNOS, pCREB and MAP kinase molecules and restored nitrite level to normal, the expression of which was altered by diazepam. The levels of cAMP, total CREB, total nitrite, nitrate and PDE were found to be unaffected by Brahmi. These behavioral findings provide tempting conclusion that Brahmi reverses amnesia induced by diazepam and can be used in the treatment of Alzheimer's Disease and Schizophrenia.

GABAergic and cholinergic system plays a vital role in reversing the amnesic behavior shown by diazepam and scopolamine. To assess the effect of Brahmi on downstream signaling molecules, amnesia was induced in mice by administrating of MK-801 and N(w)-nitro-L-arginine (L-NNA; Saraf et al., 2009). MK-801 is a NMDA receptor antagonist while L-NNA inhibits the production of nitric oxide that eventually results in memory loss. Morris water maze scale was selected to assess memory and learning skills in animals. It was found that supplementation of both of the amnesic agents in mice resulted in anterograde and retrograde amnesia and that Brahmi significantly reversed the L-NNA induced anterograde and retrograde amnesia. On the contrary, exposure of MK-1 induced mice to Brahmi didn't influence anterograde and reterograde amnesia. This suggests that Brahmi reverses the amnesic effect caused by L-NNA but not with MK-1.

**Figure 3:**
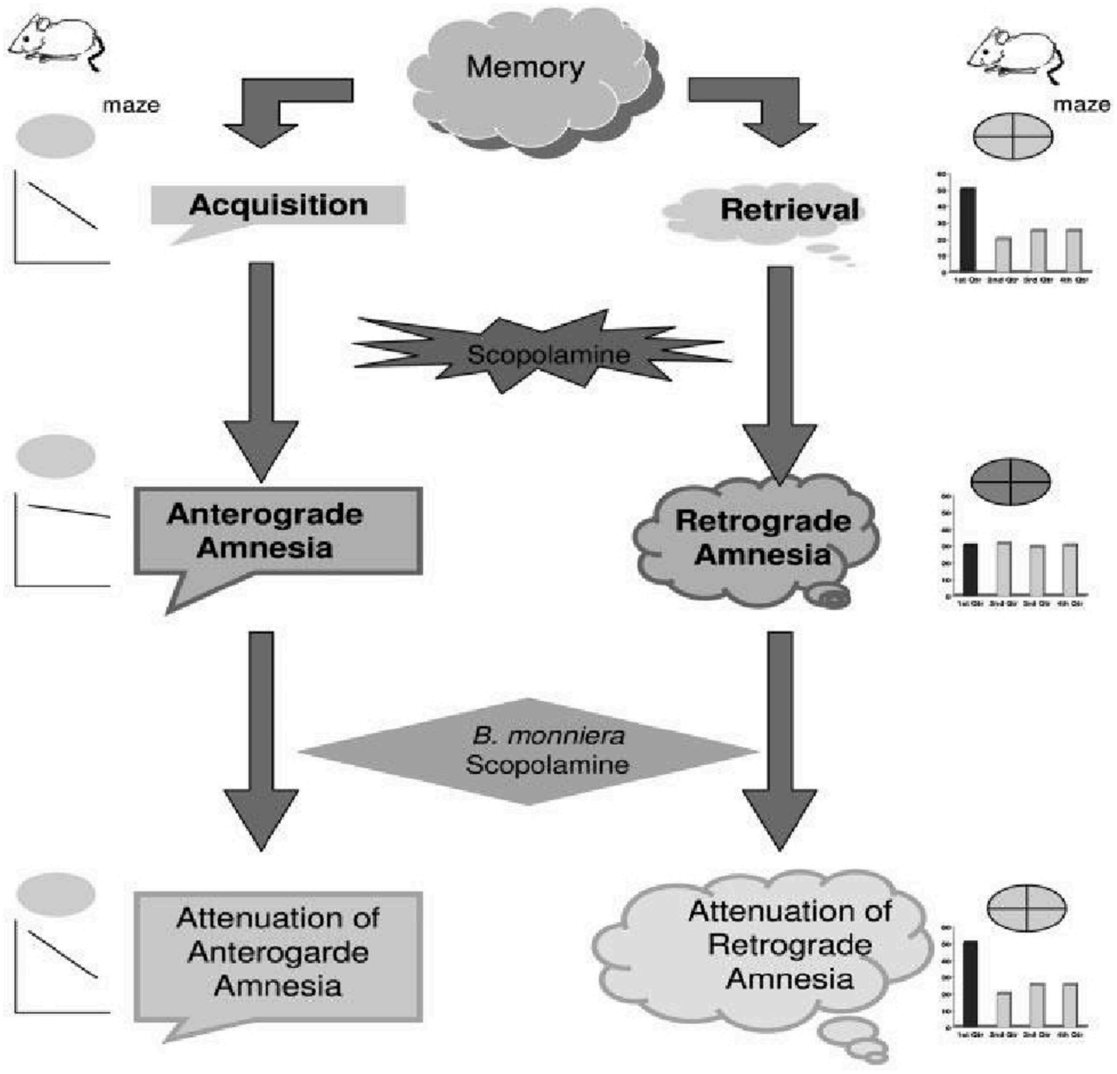
**Proposed schematic presentation of effect of *B. monniera* on acquisition and retrieval of memory**. The diagram depicts that scopolamine-induced impairment of acquisition and retrieval of memory are reversed by *B. monniera* pretreatment (Adapted from Saraf et al., 2011).

In yet another investigation, scopolamine was used to induce amnesia in mice and the effect of Brahmi was investigated on downstream signaling molecules (Saraf et al., 2010a). The findings revealed that the levels of protein kinase C and iNOS were downregulated whereas that of protein kinase A, MAP kinase, cAMP, calmodulin, pCREB, CREB and nitrite remained in normal range with the induction of scopolamine. Brahmi overruled the effect of scopolamine in amnesic mice by significantly increasing protein kinase C, calmodulin and pCREB levels. This shows that Brahmi contributes to prevent memory loss mediated by calmodulin. In addition, Brahmi is reported to significantly reverse L-NNA induced anterograde amnesia instead of MK-801 induced anterograde amnesia (Anand et al., 2010). Moreover, Brahmi ameliorates spatial memory in amnesic model of mice created using scopolamine (Saraf et al., 2011). In this study, the investigating group tested the anti-amnesic effect of Brahmi on amnesia induced by scopolamine using Morris water maze test. Muscle coordination activity in the animals was assessed using the rotarod test. The results revealed that both anterograde and retrograde amnesia produced by scopolamine were reversed by Brahmi treatment (Figure 3). These observations were suggestive of Brahmi's promising potential in the development of alternate approaches in the therapy of Alzheimer's disease. Similarly, Brahmi ameliorated memory impairment induced by phenytoin which further provides evidence for its role as an antiamnesic agent (Vohora et al., 2000). In addition, BN52021 which is a platelet-activating factor receptor antagonist was used to induce retrograde amnesia in mice was also ameliorated by Brahmi treatment. The reason for this might be an increase in glutamate level in the brain, which further increases platelet activating factor synthesis (Kishore and Singh, 2005). Brahmi also plays a neuroprotective role in rat by maintaining the level of mitochondrial enzymes, which got deviated due to morphine induction (Sumathi et al., 2011).

Recently, the neuroprotective effect of Brahmi was investigated in a rat model of schizophrenia (Piyabhan and Wetchateng, 2014). The authors evaluated discrimination ratio as a measure of cognitive ability from novel object recognition task. N-methyl-D- aspartate receptor subtype 1 (NMDAR1) density was also measured in different areas of brain including prefrontal cortex, striatum, cornu ammonis fields I (CA1) and 2/3 (CA2/3) of hippocampus and dentate gyrus (DG). The findings revealed a significant reduction in discrimination ratio in schizophrenia induced group compared to control. NMDAR1 showed upregulated expression in CA2/3 and DG but not in prefrontal cortex, striatum or CA1. Upon Brahmi administration, the DR score increased up to normal with considerable downregulation of NMDARI in CA2/3 and DG. The mechanism for memory impairment in schizophrenia induced rats appeared to be mediated by upregulation of NMDAR1 in CA2/3 and DG areas of brain. Interestingly, administration of Brahmi could restore this memory impairment by decreasing NMDAR1 in these brain areas. Therefore, Brahmi could be regarded as a novel frontier for the prevention of memory impairment in schizophrenia. The same group previously measured the vesicular glutamate transporter type 1 (VGLUT1) density in same areas of brain and found a significant decrease in the expression of this transporter of rat model of schizophrenia (Piyabhan and Wetchateng, 2013). As expected administration of Brahmi upregulated the expression of this transporter to normal level thereby proving its effectiveness in the treatment of Schizophrenia. Brahmi also possesses anti-epileptic property as evidenced by reducing the dopamine levels of dopaminergic neurons in the frontal cortex region of the rat brain (Jash and Chowdary, 2014). These observations suggest that Brahmi may possess the property to alleviate the positive symptoms of schizophrenia.

Khan et al. demonstrated the therapeutic efficacy of Brahmi on intellectual impairment and oxidative damage, induced by streptozotocin in rat models (Khan et al., 2015). The data showed that streptozotocin deteriorated memory and learning skills in these animals, which were significantly improved by Brahmi supplementation. Furthermore, increase in the amount of thiobarbituric acid reactive substances, indicative of lipid peroxidation, were visualized in the brain of these animals. Antioxidant status was also found disturbed in the hippocampus region of the brain along with a decrease in copper and zinc concentration in these experimental animals. Brahmi significantly ameliorated all of these alterations induced by streptozotocin in rats. The data suggests that formation of free radicals and increased rate of lipid peroxidation as a result of streptozotocin induction might cause neurotoxicity in these animals which can be prevented by the use of Brahmi. The study demonstrates the usefulness of Brahmi for the treatment of intellectually impaired patients.

Brahmi has been reported to increase the level of serotonin, trigger 5-HT3A receptors and CREB in hippocampus of postpartum rats thereby facilitating its learning abilities (Rajan et al., 2011). To identify the mechanism by which Brahmi acts on sodium butyrate (NaB) induced rat pups, the animals were subjected to fear conditioning test (Preethi et al., 2014). Fear conditioning is a behavioral paradigm in which an organism learns to envisage to an aversive stimulus. This stimulus is associated with neutral stimulus that elicits a state of fear response in the organism. The results revealed that Brahmi treatment in NaB induced rats facilitated the freeze response and triggered extracellular ERK/CREB signaling transduction. The levels of HAT containing coactivators such as p300, acetylated histones (e.g., Ac-H3 and Ac-H4) showed up regulated expression whereas HDACs (1, 2) and protein phosphatases (PP1α, PP2A) showed down regulated expression in hippocampus after fear conditioning test. Subsequently, the expression of brain-derived neurotrophic factor (Bdnf) (exon IV) mRNA was found to be increased indicating that Brahmi enhances hippocampus-dependent contextual memory by modulating the expression of histone acetylated proteins and protein phosphatases in hippocampus.

### Antiparkinson's effects

Apart from alleviating memory, Brahmi is demonstrated to play a role in treating Parkinson's disease, which is a neurodegenerative disorder marked by the loss of neurons which produce dopamine in substancia nigra and alpha-synuclein protein, is accumulated in the inclusion bodies known as lewy bodies (Feany and Bender, 2000). To study the mechanism underlying PD pathogenesis various experimental models have been employed. However, research on evaluating the effect of Brahmi and other plant extracts in PD models is limited. Recently, Siddique et al. investigated the effect of CDRI-08 (KeenMind) in transgenic Drosophila fruit fly (PD model) which expressed normal human alpha synuclein in their neurons. Different parameters including climbing skills, activity pattern, oxidative stress and apoptosis were measured to study the effect of Brahmi in the brain of fruit fly. Their findings revealed an improved climbing ability as well as activity pattern, reduced oxidative stress and apoptosis upon exposure of flies to Brahmi. These findings were dose dependent and suggest that the herb attenuates behavioral deformities, reduces the oxidative stress and neuronal cell death in the brains of PD model flies. Similar findings were obtained by Jansen et al., who also demonstrated the efficacy of Brahmi in alleviating the climbing activity of fruit flies compared to non-treated fruit flies (Jansen et al., 2014).

In line with previous research Jadiya et al., employed *Caenorhabditis elegans* model of Parkinson's disease to investigate the efficacy of this herb. Their findings revealed that Brahmi exposure reduced alpha synuclein accumulation, prevented dopaminergic cell death and restored the lipid content in this PD model. These data provide an evidence for Brahmi to be considered as a possible anti-Parkinsonian medication and further research on the potential use of herbal plants, compounds, and extracts in treating Parkinson's disease is required (Jadiya et al., 2011).

### Antistroke effects

The medicinal potency of Brahmi in treating Alzheimer's Disease and Parkinson's Disease has already been established. To explore its role in treating brain stroke, few investigations have been conducted by Rehni et al., who explored the role of this herbal plant on ischemia and reperfusion induced brain injury in experimental mice (Rehni et al., 2007). Ischemic-reperfusion induction lead to the increase in infarct size and impairment of short-term memory and motor balance. On the other hand, administration of Brahmi in these mice significantly reduced the infarct size and attenuated their short-term memory and motor balance. In line with this report, Saraf et al. investigated the role of Brahmi in ischemic induced brain injury in rats. Brahmi was supplemeted at a dosage of 120, 160, and 240 mg/kg in these animals and various behavioral and biochemical analysis were done to screen the efficacy of this herb (Saraf et al., 2010b). Their findings showed a protective role of Brahmi in reducing infarct size in the ischemic brain and ameliorating memory dysfunction as shown in the plus maze task. Additionally, administration of Brahmi improved the muscle coordination and catalase activity in rats exposed to ischemic insult. Levels of nitrite, nitrate and rate of lipid peroxidation were also significantly decreased. These observations indicate that Brahmi protects brain against ischemia-induced insults and further research in this direction is warranted.

Furthermore, brain ischemia reduces blood flow in cerebral arteries due to the lack of oxygen supply. To investigate whether Brahmi exerts any effect on cerebral blood flow (CBF) Kamkaew et al., measured this parameter in rats (Kamkaew et al., 2013). Rats were treated with a dose of 40 mg of Brahmi for a period of 8 weeks and thereafter, the CBF was measured via Doppler. Interestingly, the herb was found to increase the cerebral blood flow (CBF) by 25% in rats without affecting their blood pressure. These findings further confirm the efficacy of this herb in the treatment of neurological disorders and further research in this direction is required.

### Anticonvulsant effects in the treatment of epilepsy

Epileptic seizures represent a cardinal feature of the neurological syndrome named epilepsy. This disease may affect people of all ages. In recent years, an immense interest has been generated in the search of herbal drugs and formulations that may be used in the treatment of epilepsy. Brahmi is one such widely used herbal drug that alleviates nervous function, enhances memory, and reduces convulsions and inflammation. It has been reported that CDRI-08 (KeenMind) produces anticonvulsive action (Reas et al., 2008). Paulose et al., studied the role played by metabotropic glutamate-8 receptor (mGluR8) and NMDA receptor 1 (NMDAR1) gene expression in pilocarpine induced epilepsy and during neonatal hypoxia (Paulose et al., 2008). During epilepsy, mGluR8 gene was downregulated whereas NMDAR1 gene showed increased expression in hypoxic neonates. To explore the neuroprotective role of Brahmi, epileptic rats were supplemented with the herbal medication and hypoxia induced rats were supplemented with glucose, oxygen and epinephrine. The findings revealed that Brahmi treatment significantly reversed the downregulation of mGluR8 gene to normal level. Similarly, glucose supplementation together with oxygen supply in hypoxic neonates rescued the NMDAR1 gene expression to normal level. These observations suggest a neuroprotective role of Brahmi in glutamate-mediated excitotoxicity during seizures in pilocarpine-induced epilepsy. A study employed a number of convulsion inducing models including pentylenetetrazol, strychnine, hypoxic stress and pilocarpine to investigate the anticonvulsive activity of Brahmi in rats and mice. Brahmi was administered orally (50 and 55 mg/kg) in these animals, 2 and 4 h prior to receiving convulsive stimuli. It was found that the herb produced a significant anticonvulsant activity like benzodiazepines in different convulsion inducing models studied (Kaushik et al., 2009).

CDRI-08 (KeenMind) has been reported to ameliorate pilocarpine induced epilepsy through regulation of 5-HT2C and NMDA receptors in cerebral cortex (Khan et al., 2008; Krishnakumar et al., 2009a,b, 2015). During epileptic condition, the expression of glutamate receptors is altered. 5-HT2C receptor and IP3 (a signal transduction molecule) shows an elevated expression during epileptic state. On the other hand, NMDA receptor shows a downregulated expression in the brain of epileptic animals whereas mGlu5 and GLAST shows an upregulated expression leading to glutamate mediated excitotoxicity. It has been shown that treatment of epileptic rats with Brahmi reverses the changes observed in 5-HT2C, NMDA receptor expression and IP3 content thereby effectively managing the neurotransmitter balance in the cerebral cortex. These observations suggest the neuroprotective role of CDRI-08 (KeenMind) in glutamate-mediated excitotoxicity via regulating altered neurotransmitter receptor expression during seizures observed in pilocarpine induced epilepsy.

### Antidepressant effects

Brahmi appears to play a plethora of functions in the central nervous system. In addition to its diverse role in treating the diseased brain, the herb shows anti-depressant property. When mice supplemented with CDRI-08 (KeenMind) were subjected to tail suspension test (TST) and forced swimming (FST), the herbal drug exerted antidepressant activity (Shen et al., 2009). The drugs were given orally for 5 days that significantly minimized the immobility time span both in FST and TST. The antidepressant activity of Brahmi was believed to have occurred by some of its components like Bacosides A and B, bacopasaponin C, bacopasides I and II and its plant extract. However, bacopaside VII, a constituent of Brahmi, did not reveal any antidepressant activity when analyzed using tail suspension and forced swimming tests (Sairam et al., 2002; Sheikh et al., 2007; Zhou et al., 2007). Banerjee et al. investigated whether treatment with Brahmi produces any antidepressant activity in rats which were made to undergo chronic unpredictable stress based depression (Banerjee et al., 2014). The group used some behavioral tests like sucrose consumption test, shuttle box escape test and open field test to validate this hypothesis. Stress was induced in rats for a period of 4 weeks. This resulted in decreased consumption of sucrose, locomotor activity and escape latency in the animals. In addition, both mRNA and protein content of brain-derived neurotrophic factor (BDNF) showed downregulated expression in both the frontal cortex and hippocampus in CUS treated rats. Supplementation with Brahmi (80–120 mg/kg) greatly suppressed the behavioral changes and attenuated BDNF content to normal in the frontal cortex and hippocampus areas of the rat brain confirming its antidepressant activity.

The antidepressant activity of Brahmi was reported in morphine induced depression in rats (Rauf et al., 2014). Morphine, an opioid analgesic drug, when administered in rats cause depression. The drug was administered everyday two times at a dose of 20–65 mg/kg for 8 consecutive days. Three days after last morphine administration forced swimming test (FST) was conducted to assess the withdrawal effect of the drug. It was found that Brahmi treatment inhibited the withdrawal effect of the morphine induced depression. Altogether, Brahmi can be considered as a useful adjunct to cure depression like illnesses.

### Antianxiety effects

CDRI-08 (KeenMind) possess antianxiety effects, antidepressant activity, anticonvulsive action and antioxidant activity (Reas et al., 2008). Supplementation of Brahmi normalized the levels of corticosterone hormone which were imbalanced due to acute and chronic stress induction in rats. The levels of 5-HT, noradrenalin (NA) and dopamine in cortex and hippocampus regions of rats were also brought to normal in acute and chronic unpredictable stress induced animals (Sheikh et al., 2007). Brahmi modulates the cholinergic system and produces metal chelating effects. Cognitive abnormalities produced by neurotoxins, colchicine and ibotenic acid were improved by Brahmi administration in a dose dependent manner in rats (Bhattacharya et al., 2000; Rauf et al., 2012b). Also norepinephrine levels declined and 5-hydroxytryptamine levels were increased in hippocampus, cerebral cortex and hypothalamus by Brahmi treatment. Anxiety was relieved to a greater extent with higher doses of CDRI-08 (KeenMind), which was comparable to lorazepam, a standard drug used in the treatment of anxiety (Bhattacharya and Ghosal, 1998). However, treatment with lower dose of CDRI-08 (KeenMind; 10, 20, or 30 mg/kg supplemented for 1 week period) did not affect serotonin (5-HT) and dopamine levels in mice brain (Rauf et al., 2012b).

### Antioxidant effects

Oxidative stress and subsequent formation of free radicals has been implicated in the development of several diseases. Brahmi, a traditionally reputed herbal drug, has been reported to exert antioxidant activity. There are many factors that produce free radical mediated oxidative stress *in vivo*. Smoke formed from crackers increases the risk of the development of many lung diseases that ultimately results in the formation of oxidative stress. Pandareesh M. and Anand assessed whether Brahmi ameliorates the neuronal damage and physiological changes in rats upon smoke exposure (Pandareesh M. and Anand, 2014). The group exposed the rats to smoke for 1h for 3 weeks and treated the animals with Brahmi with three different dosages viz., 10, 20, and 40 mg/kg body weight. This treatment quenched reactive oxygen species formed as a result of smoke exposure and normalized the pathological changes observed in rat brain. Also, the rate of acetylcholine esterase activity, lipid peroxidation and brain neurotransmitter levels were found to be normal upon Brahmi treatment. The herb also down regulated iNOS expression thereby inhibited nitric oxide generation and HO-1 expression. Antioxidant enzyme concentration and monoamine oxidase activity were also enhanced which were depleted upon smoke exposure. These findings suggest the antioxidant and neuroprotective properties of Brahmi and may be considered as a possible remedy in the treatment of several neurodegenerative disorders.

Furthermore, oxidative stress generated by lead exposure is ameliorated by Brahmi in various areas of rat brain (Velaga et al., 2013). Lead exposure raised the levels of reactive oxygen species (ROS). Also the rate of lipid peroxidation, the carbonyl content in total protein and metal content in different tissues of rat brain. However, Brahmi treatment mitigated the levels of these proteins to normal suggesting its antioxidant property. Similarly, oxidative stress generated by sodium nitroprusside (SNP) was ameliorated by Brahmi treatment in PC12 cells (Pandareesh M. D. and Anand, 2014). In this study, Brahmi inhibited the generation of NO via down regulating iNOS expression. Heat shock proteins together with apoptotic markers such as Bax, Bcl-2, cytochrome-c and caspase-3 were also modulated to normal, the level of which was imbalanced by SNP exposure in PC12 cells. In addition, sodium nitroprusside (SNP) induced damage to plasma membrane, nuclear membrane and mitochondrial integrity of PC12 cells was ameliorated by Brahmi treatment. These findings suggests a protective and antioxidant role Brahmi exhibits to PC12 cells by mitigating the SNP induced toxicity.

Brahmi also ameliorates decabrominated diphenyl ether (PBDE-209) provoked toxicity in neonate and young female mice (Verma et al., 2014). Different doses of Brahmi (40, 80, or 120 mg/kg) in combination with PBDE-209 (20 mg/kg body weight) were administered orally in mice from postnatal day 3 to day 10. Levels of oxidative stress indicators (malondialdehyde, and protein carbonyl) and antioxidant markers (superoxide dismutase and glutathione peroxidase) were measured. The results showed that the dose of 120 mg/kg of Brahmi restored the levels of oxidants and activities of antioxidant enzymes in the hippocampus and frontal cortex of neonates against PBDE-209-induced toxicity. This data suggests that Brahmi renders the brain resistant to PBDE-209 induced toxicity and thus may be better exploited as a preventive approach to protect against oxidative-mediated neuronal dysfunctions.

Another paraquat (PQ) mediated oxidative stress and neurotoxicity was ameliorated by Brahmi treatment in different brain regions of pre-pubertal mice (Hosamani et al., 2014). Mice were administered CDRI-08 (KeenMind) daily (200 mg/kg body weight) for 4 consecutive weeks along with PQ (15 mg/kg body weight, intraperitoneally) after 3 h of last dose of extract. Within 2 days PQ administration resulted in the up regulation of oxidative stress indicating molecules (such as reactive oxygen species (ROS), hydroperoxides (HP) and malondialdehyde (MDA). However, Brahmi restored PQ induced oxidative stress back to normal via suppression of these markers in various brain regions.

Previous studies have demonstrated increased reactive oxygen species formation and cytotoxicity in cells exposed to hydrogen peroxide (H_2_O_2_). Hence, H_2_O_2_ has been greatly used to study the effects of antioxidant and cytoprotective role of herbal extracts. Pandareesh et al. studied the effect of Brahmi on H_2_O_2_mediated oxidative stress in PC12 and L132 cells (Pandareesh et al., 2014). In this study, cells were treated with H_2_O_2_ for 24 h with or without Brahmi pretreatment. A couple of tests including cell viability assay, ROS estimation, lipid peroxidation, mitochondria membrane potential assay, comet assay and gene expression studies were conducted to measure the cytoprotective activity of Brahmi. Brahmi scavenged free radicals formed during the process thereby assisting in cytoprotection. Moreover, H_2_O_2_ induced mitochondrial and plasma membrane damage was repaired by Brahmi in both of these cell lines.

Stress is a common and sometimes unavoidable problem that may lead to serious health effects. Investigators are attempting to explore the role of phytochemicals, plant extracts and compounds in modulating the activities of stress associated biomarkers. The levels of stress biomarkers namely Hsp, SOD, and cyt P450 were evaluated to study the effect of Brahmi in stress induced animals. To investigate the antistress effect, the herbal medicine was orally administered (20 and 40 mg/kg) in rats for 7 successive days (Chowdhuri et al., 2002). Stress induction elevated Hsp expression in brain. However, the protein expression remain unaltered at both doses of Brahmi in all brain regions. Interestingly, pretreatment of animals with Brahmi (7 days period) before stress induction resulted in decreased Hsp expression in all brain areas with more significant reduction in hippocampus region. At the same time, lower dose of Brahmi decreased SOD activity whereas its higher dose increased it in the hippocampus region of the rat brain. Similarly, pretreatment with a lower dose of Brahmi further reduced the SOD activity in each of the brain regions. However, higher dose of Brahmi led to elevation in the enzyme activity in all areas of the brain except cerebellum and hippocampus where it dropped significantly. Similarly stress increased the activity of Cyt P450 in all the brain regions. Almost similar results were observed with both the doses, but the P450 expression reduced with a higher dose of Brahmi. Likewise, animals pretreated with the higher dose of Brahmi followed by stress induction restored the activity of P450 enzymes to near normal levels in animals. These findings indicated the effectiveness of Brahmi in regulating stress biomarker levels and suppoting the brain to combat stressful conditions.

Mathur et al. employed DPPH radical scavenging method to investigate the antioxidant nature of four different extracts of CDRI-08 (KeenMind) in male wistar rats. It was found that all the four different extracts possessed the maximum antioxidant activity (Mathur et al., 2010). Brahmi has also been considered as a valuable supplement in the treatment of cancer and tumor. Janani et al. studied the effects of Brahmi on chemoprevention of liver cancer in an animal model (Janani et al., 2010). The group treated the animals with a carcinogen, which led to an increase in their lipid peroxidation, tumor biomarkers and markers corresponding to liver damage. Eventually, hemolysate and antioxidant status dropped markedly in the liver. Brahmi supplementation recovered the enzyme levels to normal, suppressed lipid peroxidation and enhanced its antioxidant status suggesting its chemoprotective role in the treatment of liver cancer. Similarly, the antioxidant and tumor inhibiting property of Brahmi was studied in 3-methylcholanthrene induced fibrosarcoma rats (Rohini et al., 2004). The levels of the antioxidant enzymes such as glutathione peroxidase, superoxide dismutase, catalase and glutathione, the rate of lipid peroxidation (LPO) were determined in the liver and kidney tissues. Sarcoma induction in rats resulted in a marked increase in the rate of LPO and a decrease in the antioxidant enzyme status. Furthermore, tumor markers such as lactate dehydrogenase, alanine transaminase, aspartate transaminase, creatine kinase, and sialic acid showed an upregulated expression in the serum. Brahmi supplementation enhanced the antioxidant enzyme status, reduced the rate of lipid peroxidation and downregulated tumor development markers.

Similar to many previous reports, an investigating group measured the antioxidant and lipid peroxidative status in response to Brahmi supplementation in streptozotocin induced diabetic rats. They provided CDRI-08 (KeenMind) orally to these diseased animals daily for 15 days (dosage: 50, 125, and 250 mg/kg). Thereafter, the enzymatic activity of catalase, SOD and GPx, levels of GSH and rate of lipid peroxidation were measured in kidney and brain, with special attention to cerebrum, cerebellum and midbrain. It was found that antioxidant status and peroxidative damage was totally reversed by the administration of plant extract. Activities of SOD, catalase, GPx and levels of GSH were significantly increased in diabetic rats treated with extract (Kapoor et al., 2009). To investigate the antidiabetic effect of this herb, Ghosh et al., treated alloxan induced hyperglycemic rats with CDRI-08 (KeenMind). They found that blood glucose level went down when single and multiple doses of Brahmi were provided to the animals. Weight of the rats was also recovered to normal with Brahmi treatment which was lost during diabetes. CDRI-08 (KeenMind) also inhibited the increase in glycosylated hemoglobin *in vitro* and reduced thiobarbituric acid reactive substances (TBARS) content. It also increased the levels of glutathione, SOD, catalase activity in liver of diabetic rats. The effect of extract was such that the peripheral glucose utilization was found to be increased *in vitro* in the diaphragm of diabetic rats, which was proportionate to the effect of insulin (Ghosh et al., 2010). The effects of CDRI-08 (KeenMind) (40 mg/kg) were studied on aluminum induced oxidative stress and hippocampus damage in rats (Nannepaga et al., 2014). Electron microscopy was used to evaluate any structural changes occurred as a result of aluminum intoxication in the hippocampus region of the rat brain. The enzyme activities of antioxidants such as catalase, glutathione peroxidase and SOD were determined. Aluminum administration induced oxidative damage, which was confirmed by finding increased levels of thiobarbituric acid reactive substances in the rat brain. Treatment with Brahmi for a period of 1 month decreased the levels of thiobarbituric acid reactive substances, and restored antioxidant enzyme levels. Furthermore, electron microscopy results of Brahmi treated rats revealed attenuated vacuolation, lipofuscin deposition and pyramidal cell degeneration in the hippocampus which was induced via aluminum induction. These findings further demonstrate Brahmi an important supplement useful for ameliorating the antioxidant status and inhibiting the oxidative damage occurred in aluminum intoxicated animals.

An *in vitro* study exploring the potential of Brahmi in cytokine regulation, quenching of reactive oxygen species (ROS), and ameliorating intracellular signaling pathway markers was conducted in splenocytes of F344 rats (different age groups; Priyanka et al., 2013). Firstly, concanavalin exposure induced proliferation of lymphocytes acquired from spleen of F344 rats of specific age groups (3-month-old, 8–9 month old, and 18 month old). These proliferated splenocytes were thereafter treated with different dosages of Brahmi (0.001, 0.01, 0.05, 0.1, and 1%) and donepezil (5, 10, 25, 50, and 100 μg/ml). The effect of these drugs was studied via measuring the levels of cytokines, antioxidant markers and different intracellular molecules. It was found that donepezil alone but not Brahmi reduced the lymphocyte proliferation in young rats. Cytokines such as IL-2 and IFN-γ showed elevated expression with lower doses of Brahmi whereas their expression reduced due to donepezil in rats which were young and early middle-aged. Concanavalin exposure also reduced the activity of antioxidant markers (SOD, CAT, GPx, and GST) in these cells. On the contrary, enhancement in CAT activity was observed in all age groups with Brahmi supplementation whereas in older rats donepezil increased the SOD activity. Likewise the action of GPx and GST biomarkers was enhanced in lymphocytes via both Brahmi and donepezil administration all age groups. Whereas in splenocytes there was an enhancement in the rate of lipid peroxidation and age-related suppression in NO generation. Brahmi along with donepezil raised up NO production in the lymphocytes of early middle-aged and older rats. It is important to note that donepezil could suppress lipid peroxidation splenocytes of only old rats whereas Brahmi could do the same in both early-middle-aged and old rats. Similarly the reversal in decline of the p-Akt expression was achived by Brahmi in both early middle aged and old rats' lymphocytes. We can conclude that Brahmi alongwith donepezil exert various age-related effects on cytokine generation, antioxidant status and intracellular targets which may further manipulate the therapeutic efficiency of these drugs in various diseases.

### Gastrointestinal and hepatoprotective effects

Brahmi has been shown to cure a number of gastrointestinal disorders. A dose of 500 mg/kg of CDRI-08 (KeenMind) when supplied orally prevented the development of diarrhea in mice. As compared to loperamide (50 mg/kg) Brahmi was able to decrease the frequency of defecation suggesting its role as an anti-diarrhoeal herb (Siraj et al., 2012). Also, the fresh juice of this herbal medicine has been shown to prevent the formation of ulcers (Rao et al., 2000). In this study, the group assessed the prophylactic and healing effects CDRI-08 (KeenMind) exerts in five different models of gastric ulcers. It was found that the extract (20 mg/kg for 10 consecutive days) alleviated acetic acid induced penetrating ulcers, strengthened mucosal barrier and reduced mucosal exfoliation by reducing the rate of lipid peroxidation in gastric mucosa of rat. The extract has also been reported to produce anti-Helicobacter pylori activity (Sairam et al., 2001; Goel et al., 2003). A randomized, double-blind, placebo controlled clinical trial of 169 irritable bowel syndrome patients, was conducted to assess the therapeutic capacity of an Ayurvedic preparation containing Brahmi and *Aegle marmelos* herbs. These herbal drugs were provided orally to the patients three times daily for a period of 6 weeks. The effect of ayurvedic treatment was highly beneficial in curing the disease. However, the extent of Brahmi's efficacy could not be predicted as both the drugs were given at the same time to the patients (Yadav et al., 1989).

CDRI-08 (KeenMind) has been found to exert hepatoprotective effect in rats by alleviating antioxidant enzymes status induced by morphine (Sumathi et al., 2011). It was found that morphine induction increased the rate of lipid peroxidation and decreased antioxidant enzyme status in rats. This toxicity induced as a result of morphine induction was nullified by the supplementation of Brahmi.

### Endocrine effects

The potential of Brahmi in treating endocrine related abnormalities has been documented in a few animal studies. CDRI-08 (KeenMind; 200 mg/kg orally) has been shown to alter the secretion of thyroid hormone in male mice (Kar et al., 2002). The synthesis of T4 hormone was increased by 41% via Brahmi intake. However, T3 synthesis was unaffected by the drug supplement indicating that the drug might not be involved in T4 to T3 conversion. Furthermore, Brahmi has been demonstrated to possess the anti-fertility ability in male mouse (Singh and Singh, 2009).

### Antimicrobial effects

Methanolic extracts of CDRI-08 (KeenMind) have been reported to possess antimicrobial activity as compared to other extracts (Azad et al., 2012; Katoch et al., 2014). In this study, hexane and petroleum ether extracts inhibited the growth of microbes in a similar manner but the effect was less considerable in comparison to methanolic extracts whereas the aqueous extract of CDRI-08 (KeenMind) did not show anti-microbial activity (Hosamani et al., 2014). The growth of Staphylococcus aureus was inhibited to a greater extent by the methanolic extract (1mg/ml) of CDRI-08 (KeenMind) in comparison to Salmonella typhi and Eschirichia coli. However, the extract did not suppress the growth of K. pneumonia microbe (Rohini et al., 2004).

### Anti-inflammatory and painkiller effects

Brahmi, known for its anti-inflammatory and pain relieving effects, acts by selectively inhibiting cyclo-oxygenase-2 enzyme, and consequently reducing prostaglandins synthesis. Jain et al. demonstrated that CDRI-08 (KeenMind) could effectively suppress the experimentally produced inflammatory reaction by quenching the synthesis of prostaglandins and preventing lyosomal membranes from rupture. Also, treatment with anti-inflammatory dose of Brahmi didn't cause any gastric problems (Jain et al., 1994). Writhing produced by acetic acid in mice was reduced using whole plant ethanol extract of CDRI-08 (KeenMind) (250 and 500 mg/kg; Rao et al., 2000). Mathur et al. used different extracts of CDRI-08 (KeenMind) and studied their anti-inflammatory effects in edema caused by carrageenan in rat's hind paws. Supplements including methanolic and aqueous extract (100 mg/kg) of Brahmi were found to significantly reduce inflammation, while, petroleum ether and hexane extracts produced no effect (Janani et al., 2010). Also, it was found that methanolic extract (100, 200, 300 μg) assisted in membrane stabilization as compared to diclofenac sodium (Rohini et al., 2004). Interleukin-6 and tumor necrosis factor-alpha synthesis was inhibited by the fractions of Brahmi containing triterpenoids and bacosides (Viji and Helen, 2011). Furthermore, proinflammatory cytokines such as nitric oxide and TNF-α showed down regulated expression in stimulated macrophages and IFN-γ in stimulated human blood cells (Williams et al., 2014). These results provide further evidences that confirm the efficacy of Brahmi in the treatment of brain inflammation.

### Relaxant effects on smooth and cardiac muscles

Investigations exploring the potential of CDRI-08 (KeenMind) in relaxing cardiac and smooth muscles have been demonstrated in experimental animals. The herbal extract ameliorates left ventricular contractility, coronary blood flow and heart rate in rabbit's heart (Rashid et al., 1990). It also relaxes bronchial smooth muscles, pulmonary arteries, aorta, and trachea. Apparently, the mechanism of action Brahmi exerts on the cardiac muscles is quite similar to that of quinidine. These effects possibly were mediated by accumulation of calcium ions in the extracellular space (Dar and Channa, 1997, 1999; Channa et al., 2003). Furthermore, CDRI-08 (KeenMind) stabilizes the activity of mast cells comparable to disodium cromoglycate (Samiulla et al., 2001).

## Clinical trials

Traditionally Brahmi is known to ameliorate cognitive function. This viewpoint has now been scientifically tested through a handful of randomized, double-blind, placebo-controlled clinical trials and nearly all have shown promising results. (Singh et al., 2014) found that supplementation of 12g of Brahmi improved the nervousness, concentration and memory in adults. The dose was given to 35 adults in the form of syrup for 4 weeks. There was no complaint of side effects (Singh and Singh, 1980). Similar observations were confirmed by Sharma et al. who studied the effect of Brahmi in 20 primary school children (Sharma et al., 1987). A dosage of 350 mg was given in syrup form three times a day for 3 months. The herb improved learning skills, perception, memory and reaction times in them without the occurrence of any side effects. A randomized and double-blind placebo-controlled trial in 36 children was conducted, who were affected with attention deficit hyperactivity disorder (ADHD) was conducted (Negi et al., 2000). The results were highly beneficial with Brahmi supplementation as it greatly improved the logical memory. In this study, freshly preperaed whole plant extract of Brahmi was administered at a dosage of 50 mg two times a day for a period of 12 weeks. Later, cognitive function tests were performed at various time points which included baseline, 4, 8, 12, and 16 weeks. Recovery was observed in the 12 weeks group, consolidated by different cognitive tests. A placebo controlled study demonstrated enhanced learning and controlled abnormal behavior in 40 mentally retarded children consuming standardized extract of CDRI-08 (KeenMind; Dave et al., 1993). Another randomized, double-blind, placebo-controlled trial proved the effectiveness of Brahmi in ameliorating memory (Roodenrys et al., 2002). In this study, 76 healthy adults, 40–65 years old, were supplemented with Brahmi (dose 300 mg) and they all were benefitted by retaining the information in delayed recall of word pairs. However, there were a few parameters which failed to show the beneficial effect of Brahmi. These include attention, working memory, short-term memory tasks, psychological state and retrieval of prior knowledge. Likewise, no effect of Brahmi (300 mg dosage) on various measures of memory performance was found when it was administered 2 h after treatment suggesting that its benefits are obtained after long-term use (Nathan et al., 2001). The same group of investigators later found no significant effect of Brahmi (300 mg/day) on cognition and memory when provided in combination with *Ginko biloba* 120 mg/day for a period of 4 weeks. This was a randomized, placebo-controlled, double-blinded clinical trial, conducted in 85 healthy subjects (Nathan et al., 2004). Bacomind™ capsule when consumed orally (at a dose of 300 mg once a day for first 15 days and 450 mg once a day for next 15 days) improved mental functioning in 23 healthy adults (Pravina et al., 2007).

A placebo-controlled and double-blinded 12-week clinical trial was carried out to investigate the effectiveness of CDRI-08 (KeenMind) (300 mg daily for 12 weeks) on 46 healthy people aged between 18 and 60 years. A series of cognition function tests were conducted at baseline and later on after 5 and 12 weeks. At the end of 12 weeks, in the treatment group a significant enahncement in verbal learning and concentration was noticed compared to non-treated groups. These effects were not observed at baseline or at 5 weeks of treatment (Stough et al., 2001). Likewise many other clinical trials were conducted to determine the effect of Brahmi on memory function in elderly people above the age of 55 years. They all used same criteria with same amount of dosage and period of administration. It was found that aged people were able to acquire, store and retain their memory over time by consuming Brahmi as a supplement (Calabrese et al., 2008; Morgan and Stevens, 2010). Another randomized double-blind placebo-controlled clinical trial examining the effect of Brahmi on cognitive, biochemical and cardiovascular performance was conducted in elderly people (Morgan and Stevens, 2010). This study involved a large number of randomly selected participants (465 participants) aged between 60 and 75 years. CDRI-08 (KeenMind) (300 mg/day) was given to them for a period of 12 months. The participants underwent a series of cognitive function tests at points: baseline, 3, 6, and 12 months and expectedly, the results revealed a significant improvement in their memory function which suggests the effectiveness of Brahmi to be used as a memory booster.

Peth Nui et al. tested different aspects of brain function like attention, cholinergic and monoaminergic functions, memory processing and working memory-using Brahmi. They recruited 60 healthy adults aged around 60 years for a randomized, double-blind, placebo-controlled clinical trial. The period of Brahmi administration and dosage was similar to previous studies (300 mg for 12 weeks; Peth-Nui et al., 2012). AChE and MAO activities were measured to evaluate the cholinergic and monoaminergic systems functions. On the contrary, percent accuracy and reaction time was examined to determine the working memory. Latencies and amplitude of N100 were used to measure attention and cognitive processing. The findings of this study revealed a reduction in both N100 and P300 latencies and improvement on working memory with Brahmi supplementation. Furthermore, AChE activity was also found to be decreased suggesting that Brahmi can ameliorate cognitive processing, working memory and attention partly through the reduction of AChE activity. In another similar kind of clinical trial involving healthy elderly subjects and others suffering from senile dementia of Alzheimer's type (SDAT), behavioral and biochemical parameters like learning abilities, inflammatory markers and oxidative stress were measured (Sadhu et al., 2014). A number of cognitive function tests were employed after every 3 months to evaluate the potential of Brahmi in these people. It was found that Brahmi treated SDAT patients improved memory performance when compared to controls. The levels of inflammatory markers like homocysteine, C-reactive protein, and tumor necrosis factor alpha; oxidative stress markers like glutathione peroxidase, glutathione, thiobarbituric acid reactive substances and SOD showed a marked decline in Brahmi treated SDAT patients. This suggests the significance of the herb in managing cognitive decline associated with the aging process. Another multicenter clinical trial involving patients with mild cognitive impairment used similar criteria and obtained favorable results establishing the role of Brahmi in improving cognitive function (Zanotta et al., 2014).

Recently, the acute effects of Brahmi (320 and 640 mg doses) on stress and mood swings generated by multitasking were demonstrated in a double-blind, placebo-controlled clinical trial involving 17 healthy volunteers (Benson et al., 2014). Brahmi supplementation reduced stress as observed by reduction in cortisol levels and alleviated mood in these participants. Altogether, these studies demonstrate the therapeutic efficacy of Brahmi in alleviating various abnormalities hence can be considered as a promising frontier to treat various diseases. Table 1 indicates clinical trials of Brahmi.

**Table 1:** **Clinical trials of Brahmi**.

**Study design**	**Subjects**	**Dosage of Brahmi/day**	**Time span of the drug administration**	**Results**	**References**
–	35 adults	12 gm/day	4 weeks	Reduced anxiety, enhanced memory span and concentration. No side effects observed.	Singh and Singh, 1980
–	20 primary school children	350 mg × 3/day	3 months	Enhanced memory, learning, perception and reaction times. No side effects observed.	Sharma et al., 1987
PC	40 Mentally retarded children with or without epilepsy	CDRI-08 (KeenMind)	–	Enhanced learning and controlled abnormal behavior.	Dave et al., 1993
RA, DB, PC	36 children with Attention deficit hyperactivity disorder (ADHD)	50 mg × 2/day	12 weeks	Ameliorated various cognitive assessments. No side effects observed.	Negi et al., 2000
–	38 healthy subjects	300 mg/day	2 h post administration	No improvement in memory performance.	Nathan et al., 2001
DB, PC	46 healthy people	300 mg/day	12 weeks	Improved early information processing and verbal learning rate. Consolidated memory and reduced state anxiety. Side effects: nausea, dry mouth and fatigue.	Stough et al., 2001
RA, DB, PC	76 healthy adults	300 mg/day	12 weeks	Enhanced retention of new information.	Roodenrys et al., 2002
RA, DB, PC	85 healthy subjects	Combination of standardized CDRI-08 (KeenMind) 300 mg/day and *Ginko biloba* 120 mg/day	4 weeks	No significant effect on cognition and memory.	Nathan et al., 2004
RA, OL, DE	23 healthy adult volunteers	Bacomind™ capsule 300 mg/day and 450 mg	15 days each, respectively	Improved cognition. Minor gastrointestinal adverse effects.	Pravina et al., 2007
RA, DB, PC	54 healthy adults	Standardized CDRI-08 (KeenMind) 300 mg/day	12 weeks	Enhanced cognitive performance in the aging.	Calabrese et al., 2008
RA, DB, PC	98 healthy adults	Bacomind™ capsule 300 mg	12 weeks	A significant improvement in memory acquisition and retention was observed. Gastrointestinal side effects reported.	Morgan and Stevens, 2010
RA, DB, PC	465 participants	300 mg/day	12 months	Improvement in memory function.	Stough et al., 2012
RA, DB, PC	60 healthy adults	300 mg/day	12 weeks	Attention, cognitive processing, and working memory improved.	Peth-Nui et al., 2012
RA, DB, PC	109 healthy subjects and 123 SDAT patients	500 mg × 2/day	12 months	Improvements in memory performance and reduction in the levels of inflammatory and oxidative stress markers observed in Brahmi treated SDAT patients.	Sadhu et al., 2014
PC, NC, MC	104 elderly subjects with mild cognitive impairment	1 Illumina® tablet/day	60 days	Cognitive function improved. One non serious adverse effect reported.	Zanotta et al., 2014
DB, PC	17 healthy volunteers	320 mg and 640 mg		Brahmi supplementation reduced stress and alleviated mood in these participants.	Benson et al., 2014

RA, Randomized; DB, Double-blind; PC, Placebo-controlled; OL, Open label; DE, Dose escalation; PC, Prospective cohort; NC, Noncomparative; MC, Multicenter.

## Future directions

The therapeutic effects of Brahmi have been extensively investigated by various research groups. It is believed that the bacosides, act as active constituents of the herbal extract which are predominantly involved in exerting the nootropic effects in both animals and humans. The delivery of active components to brain itself is a challenging task, which can be solved to great extend by nanoscience. As nanoscience has emerged as a subject of significant curiosity which associated with special properties like surface to volume ratio and surface reactivity. Drug delivery to the brain represents one of the most important challenges in the field of nanomedicine. At the same time, a better understanding of the physiopathological nature of different diseases associated to cognitive science and insight into the interaction of nanomaterials with biological systems at various levels (i.e., systemic, organ, tissue, and cell) are of paramount importance for further progress toward bench-to-bedside translation or the mechanisms underlying the effects of Brahmi in various disease conditions need to be elucidated extensively at the molecular level and followed by clinical trials. In order to make the extract/ active components targeted to brain, use of targeted nanoparticles based on liposomes, polymeric micelles, polymersomes can be considered as an important tool.

## Author contributions

DM and KG wrote the manuscript. AA and VK conceptualized and edited the manuscript.

### Conflict of interest statement

The authors declare that the research was conducted in the absence of any commercial or financial relationships that could be construed as a potential conflict of interest.
